# Existential priming in L2 Chinese: proficiency-selective modulation and locus-specific lexical boost

**DOI:** 10.3389/fpsyg.2026.1716848

**Published:** 2026-01-29

**Authors:** Jinxin Qian, Xiao Cheng, Jiayuan Yu

**Affiliations:** 1 Department of International Cultural Education, Nanjing Normal University, Nanjing, China; 2 Department of Psychology, Nanjing Normal University, Nanjing, China

**Keywords:** L2 proficiency, lexical boost, mandarin existential sentences, preference effects, structural priming

## Abstract

Structural priming offers a window on how L2 speakers retrieve and assemble grammatical knowledge, yet evidence from Mandarin existential templates remains limited. We report two production experiments with L2 learners. In Experiment 1, participants completed a picture description task targeting three existential patterns: EX-ZHE (Location +V + 着 + NP), EX-LE (Location + V + 了 + NP), and EX-YOU (Location + 有 + NP). A binomial mixed-effects model of existential realization (existential vs. non-existential output) showed reliable priming, strong prime-type differences, and a Prime Type × Proficiency interaction: EX-ZHE and EX-YOU primes increased existential production, whereas EX-LE reduced it; proficiency increased existential realization overall but most clearly following EX-ZHE primes. Experiment 2 fixed EX-ZHE as the target and manipulated prime–target lexical overlap (verb, sentence-initial locative, post-verbal noun, or no overlap). Lexical boost was locus-dependent: verb overlap produced the largest facilitation, locative overlap also increased EX-ZHE realization, and post-verbal noun overlap did not reliably differ from baseline; proficiency did not modulate these effects. Together, the findings support a cost and cue-sensitive account in which constructional availability and diagnostic lexical retrieval cues jointly shape L2 alignment. Any instructional implications are treated as hypotheses from controlled tasks and require corpus- and classroom-based validation.

## 1 Introduction

### 1.1 Background of study

Structural priming refers to the tendency for speakers to reuse a recently processed syntactic structure in subsequent language production (Bock, 1986). Since its seminal demonstrations in sentence production (Bock, 1986; Bock, 1989, 1990) and the subsequent extension to a range of constructions (Bock, 1989; Branigan et al., 2000; Pickering and Branigan, 1998), structural priming has become a core experimental tool for probing the mental representation of syntactic knowledge and the mechanisms that support fluent sentence generation. At a basic descriptive level, the phenomenon is robust across tasks and modalities (e.g., spoken vs. written production) and can persist across intervening material, with effect sizes modulated by linguistic and cognitive factors (Branigan et al., 2005; Mahowald et al., 2016).

However, the field has gradually moved from documenting “whether priming exists” to explaining what exactly is primed, where priming lives in the language system, and why priming sometimes strengthens or disappears. Three interlocking research strands are particularly central to a systematic review.

First, structural priming is theoretically informative because it constrains the format of syntactic representation. Classic accounts have emphasized abstract syntactic frames and their reactivation in later production (Pickering and Branigan, 1998; Branigan et al., 2000; Cleland, 2003). Yet a persistent complication is that priming is rarely “purely syntactic”: lexical repetition, event structure overlap, and discourse reuse can all mimic or magnify structural persistence. Corpus-based work has underscored this point by showing that priming effects are entangled with distributional properties of individual verbs and construction frequencies, and that “verb-specific sensitivity” may be hidden when experiments do not adequately control baseline probabilities (Gries, 2005). This motivates a key methodological principle for modern priming research: to interpret priming as evidence for abstract structure, one must control (or model) lexical biases and baseline frequencies rather than assume they cancel out. More broadly, rather than treating syntactic structure as independent of lexis, we adopt an interface perspective in which construction choice is shaped by lexicalized constraints and cue-based retrieval. Lexical priming work shows that priming effects can be restricted to argument preferences and other lexical–grammatical contingencies (Novick et al., 2003)., and usage-based accounts similarly emphasize learned word–construction regularities that can be reactivated as part of priming (Schmid, 2007; Ellis et al., 2009). Accordingly, our two-experiment design tests both construction-level persistence (Experiment 1) and locus-specific lexical boost (Experiment 2) within Mandarin existential templates, directly addressing how lexical cues interface with apparent structural alignment.

Second, the mechanisms of priming remain debated, and the debate is not cosmetic—it affects what counts as a contribution. On one side, residual activation views priming as transient carryover of recently activated combinatorial nodes or structural frames (Pickering and Branigan, 1998). On the other side, implicit learning accounts propose that priming reflects experience-driven adjustment of production probabilities, yielding potentially longer-lasting changes (Chang et al., 2006, 2012; Reitter et al., 2011). A large-scale synthesis suggests that different paradigms and time scales may recruit these mechanisms to different degrees (Mahowald et al., 2016), which helps explain why some studies find short-lived priming while others observe more durable adaptation. Importantly, the theoretical payoff of a new priming study depends on whether the design can adjudicate between (or at least constrain) these mechanisms, for example by manipulating lag, repetition, or cumulative exposure.

Third, a central set of moderators—especially relevant for L2 and for under-studied constructions—concerns lexically mediated boosts, proficiency, and preference. Many studies report a “lexical boost,” i.e., larger priming when prime and target share a lexical head (often the verb) (Pickering and Branigan, 1998; Cleland, 2003). Yet subsequent work shows that this effect is not uniform and depends on which lexical element repeats and how strongly that element anchors structure (Arai et al., 2007). Recent evidence suggests that verb repetition yields a more reliable boost than non-verb repetition, aligning with the idea that verbs provide combinatorial constraints and are central to argument-structure encoding (Hartsuiker et al., 2008). Relatedly, research beyond classic sentence-level priming converges on the broader principle that different constituents contribute asymmetrically to priming, depending on their structural roles. For instance, work on noun–noun combinations demonstrates stronger lexical priming from head nouns than from modifiers, while relational priming patterns may track different representational loci (Gagné, 2001). This asymmetry matters for syntactic priming because it predicts that repeating a “head-like” element (e.g., a verb or a structurally privileged noun) may not be equivalent to repeating a peripheral element, even if both are lexically identical. However, evidence for non-head repetition is mixed: some studies report measurable boosts from repeating non-verbal constituents, whereas others find that such effects are weak, contingent, or absent once baseline preferences and structural competition are appropriately modeled (Scheepers et al., 2017; Carminati et al., 2019; Guimarães and Silva, 2021; Van Gompel et al., 2023). Related debates about head vs. non-head contributions and preference-sensitive patterns further suggest that priming magnitude and direction can depend on representational accessibility and competition among alternatives (Segaert et al., 2014; Traxler et al., 2014; Kantola et al., 2023).

In parallel, priming has also been discussed within broader “priming architectures” that connect lexicon, grammar, and experience. Usage-based perspectives argue that speakers store rich distributional regularities linking words, constructions, and discourse contexts, and that priming reflects the reactivation of these learned patterns (Schmid, 2007).

From this angle, what looks like “structural priming” may sometimes be the reuse of entrenched multi-word patterns or chunks. Evidence consistent with chunk-sensitive planning exists even in production domains such as writing, where temporal signatures can reveal phrase-level chunking during formulation (Cheng and Rojas-Anaya, 2006). This connects naturally to L2 production, where planning scope and chunk retrieval may differ from L1 and therefore interact with priming magnitude.

A further dimension concerns cross-linguistic and bilingual priming, which speaks directly to whether syntactic representations are shared across languages. Cross-linguistic priming of syntactic ambiguity resolution has been shown for hierarchical configuration information not directly tied to lexical entries, supporting shared-syntax accounts (Desmet and Declercq, 2006). More broadly, bilingual and L2 priming has been used to probe representational sharing and proficiency-related modulation across languages, though patterns vary across tasks and populations (Schoonbaert et al., 2007; Zhao et al., 2011; Bernolet et al., 2013; Jackson and Ruf, 2017; Coumel et al., 2021).

This line of work is crucial for L2 priming studies because it implies that priming can index representational overlap even when lexical overlap is limited—provided that the design targets genuinely structural properties.

Taken together, the literature supports an integrated framing of structural priming research around four interlocking questions. A first question concerns what is primed: whether priming primarily reflects abstract structural frames or structures that are tightly tethered to lexical representations, and whether priming is disproportionately carried by head elements relative to non-head constituents. A second question concerns how priming is implemented in the production system: whether observed persistence is best captured as short-lived residual activation, as experience-driven implicit learning, or as a combination in which short-term boosts coexist with longer-run adaptation. A third question concerns what moderates priming, including lexical overlap, proficiency, preference/baseline frequency, and planning scope—factors that can amplify, attenuate, or even reverse priming depending on the construction and task. A fourth question concerns how we know, namely the methodological requirements for identifying priming as evidence about representation and mechanism: controlling or modeling verb bias, construction frequency, and discourse reuse, and triangulating experimental evidence with corpus-based baselines where possible.

This integrated view also clarifies why “scarcity of studies on a construction” is not, by itself, a sufficient gap statement. A compelling gap must specify which of these questions remains theoretically ambiguous for the target construction and why that ambiguity matters. In the present case, Mandarin existential templates offer a particularly diagnostic testbed because they couple construction choice with aspectual morphology and form–meaning mapping demands, making it possible to ask (i) whether priming reflects abstract persistence versus lexically supported retrieval, (ii) how cost and representational stability constrain priming across templates, and (iii) whether lexical boost effects concentrate at cue-diagnostic loci under varying proficiency. These issues are especially salient in L2 production, where proficiency and distributional preference have been argued to modulate alignment yet show mixed evidence across studies (Ferreira and Bock, 2006; Ameri-Golestan and Nezakat-Alhossaini, 2013; Segaert et al., 2016; Wei et al., 2022).

### 1.2 Current study

Despite the extensive priming literature in Indo-European languages, syntactic priming research in Chinese—especially in L2 acquisition—has focused disproportionately on a limited set of constructions and has often inherited unresolved debates without directly addressing them (Ferreira and Bock, 2006; Zhao et al., 2011). For example, proficiency has been found to modulate priming in some contexts (Ameri-Golestan and Nezakat-Alhossaini, 2013; Segaert et al., 2016), but results are mixed: some studies report strong priming even in low-proficiency learners, whereas others observe increasing priming with proficiency (Wei et al., 2022). Likewise, “inverse preference effects” (stronger priming for less preferred structures) have been argued to reflect learning-based adaptation (Chang et al., 2006, 2012; Reitter et al., 2011), but competing findings show preference-sensitive patterns that can be interpreted through activation-based or distributional accounts (Segaert et al., 2014; Traxler et al., 2014). These inconsistencies are not merely empirical noise; they signal that priming effects may conflate structural availability, lexical biases, and baseline probabilities—exactly the confounds highlighted in corpus-oriented critiques (Gries, 2005).

Within Chinese, existential sentences constitute a particularly diagnostic testbed because they combine (i) constructional alternations, (ii) aspect marking, and (iii) verb–argument packaging constraints that can shift both preference and processing load. Yet L2 priming studies on Chinese existential constructions remain scarce (Zhang and Wang, 2012; Kong and Zou, 2022; Zha and Wu, 2014; Zhang and Wang, 2013; Zuo, 2017), even though existential sentences are central to everyday discourse and encode a distinctive mapping between location, existence, and event structure. In Chinese, existential sentences typically place the locative phrase sentence-initially and present the figure/entity post-verbally, as in:

1. 桌子上放着一本书。
2. 桌子上放了一本书。
3. 桌子上有一本书。

These patterns differ not only in surface form but also in aspectual and informational properties. Theoretical descriptions emphasize that existential “有” differs in semantic-pragmatic profile from existential uses involving a lexical verb plus aspect marker “着/了” (Lei, 1993). Such contrasts create an opportunity to test whether priming targets abstract structural templates, aspectual feature bundles, or lexically anchored mappings.

Against this background, the present study is designed to address specific limitations of prior syntactic priming research, rather than merely adding “another construction.” Concretely, previous priming work leaves at least three unresolved issues that are especially acute for existential sentences:

1. Separating structure from baseline preference and verb bias. Much experimental priming research assumes that priming reflects structural repetition, but corpus and distributional work warns that constructional choices are strongly conditioned by verb-specific and frequency-driven preferences (Gries, 2005). Existential alternations in Chinese, with their aspectual and lexical constraints, require explicit attention to baseline preferences and lexical distributions to avoid misattributing frequency-driven repetition as structural priming.
2. Locating the lexical boost in non-canonical constructions. The lexical boost is often treated as “verb repetition boosts structure,” yet its magnitude depends on which element repeats and whether that element functions as a structural anchor (Pickering and Branigan, 1998; Arai et al., 2007; Hartsuiker et al., 2008). Evidence from other priming domains suggests head-like elements can drive stronger priming than non-head elements (Gagné, 2001). Existential sentences allow a sharper test of this question because potential repeating elements (locative phrase, verb, postverbal NP) differ in their structural roles.
3. Connecting proficiency and inverse preference effects to mechanism-level theories. Prior L2 studies have reported proficiency effects but remain theoretically under-integrated, rarely leveraging the residual activation vs. implicit learning debate to interpret why proficiency modulates priming (Ameri-Golestan and Nezakat-Alhossaini, 2013; Segaert et al., 2016; Wei et al., 2022). Similarly, preference effects have been invoked but not consistently tied to learning-based predictions (Segaert et al., 2014; Traxler et al., 2014). Because existential alternations involve markedness and distributional asymmetries, they provide a principled context to test whether priming aligns better with transient activation (stronger when representations are readily available) or with implicit learning (stronger adaptation for less expected structures).

By targeting these issues, the current study aims to make two broader contributions. Empirically, it expands the typological and constructional scope of L2 syntactic priming by examining Chinese existential structures, which have been comparatively neglected. Theoretically, it uses this constructional domain to tighten the link between observed priming patterns and competing mechanism-level accounts (residual activation vs. implicit learning), while explicitly considering lexical anchoring, baseline preferences, and distributional constraints. In doing so, the study also speaks to usage-based perspectives that view priming as the reactivation of experience-shaped lexical–grammatical patterns (Schmid, 2007) and to bilingual priming evidence suggesting that structural information can be primed even when not directly tied to lexical entries (Desmet and Declercq, 2006).

## 2 Experiment 1

Experiment 1 tested whether L2 speakers of Chinese exhibit structural priming for Mandarin static existential constructions and whether priming is moderated by (i) construction type and (ii) proficiency, with particular attention to preference vs. inverse-preference patterns. This design is theoretically diagnostic in two respects. First, existential variants (EX-ZHE/EX-LE/EX-YOU) differ in aspectual marking and form–meaning mapping, allowing priming to be assessed in a construction family where structural choice is not merely a surface alternation. Second, because L2 learners are known to over-rely on the default-like existential 有, the existential domain provides a principled test of whether priming is stronger for preferred (high-baseline) forms or shows an inverse-preference pattern consistent with learning-based adaptation (Chang et al., 2006; Segaert et al., 2014; Mahowald et al., 2016).

Accordingly, Experiment 1 addressed three questions:

(1) Is there a syntactic priming effect in the production of existential sentences by L2 Chinese learners? (2) If there is a priming effect, are there significant differences in the production of target structures between participants at different levels of Chinese proficiency and for different types of prime sentences? (3) How do L2 Chinese learners exhibit the structural priming effect for existential sentences, and does this priming show a preference or reverse preference effect?

### 2.1 Method

#### 2.1.1 Participants

To determine an appropriate sample size, we conducted an *a priori* power analysis in G*Power 3.1.9.7 using repeated-measures ANOVA as a conservative approximation (power = 0.80, *α* = 0.05, reference effect size *f* = 0.40), which suggested a minimum of 64 participants. Following this estimate, and considering typical sample sizes used in syntactic priming research and recommendations discussed in meta-analytic work (Mahowald et al., 2016), we recruited 105 participants for Experiment 1. The same sample size was used in Experiment 2 given the comparable design and procedures.

A total of 105 international students studying Chinese at the School of International Cultural Education at Nanjing Normal University participated. All participants’ first language (L1) was English. Mean age was 20.5 years (SD = 1.23). L2 proficiency was indexed by participants’ average scores across five simulated HSK Level 4 tests (treated as a continuous proficiency variable in subsequent analyses). Participants received small gifts as compensation and reported no reading or speaking impairments.

#### 2.1.2 Materials

Experiment 1 employed a picture-description paradigm. The target structures were three Mandarin static existential patterns: EX-ZHE (Location + V + 着 + NP), EX-LE (Location + V + 了 + NP), and EX-YOU (Location + 有 + NP). Each type was represented by four prime sentences. Fifteen HSK Level 4 verbs known to participants were used across the existential sentences: 停, 挂, 摆, 放, 拿, 躺, 站, 坐, 写, 画, 贴, 种, 戴, 堆, 开, 装. The experimenter (participants’ course instructor) confirmed that all depicted objects and relevant lexical items were familiar to participants.

The full stimulus set included 54 pictures: 12 experimental prime pictures with sentences (sentence displayed below the picture; read aloud), 12 experimental target pictures (no text; described by participants), and 30 fillers. Both prime and target pictures contained two core elements: a locative noun (e.g., floor, table, wall) and an entity/object (e.g., shoe, flower, clothing). Target pictures were intentionally underspecified for structure, allowing either an existential description or alternative constructions (e.g., “That is clothing”/“Whose clothing is this?”), so that priming could be observed as a shift in structural choice rather than forced repetition.

**Figure 1:**
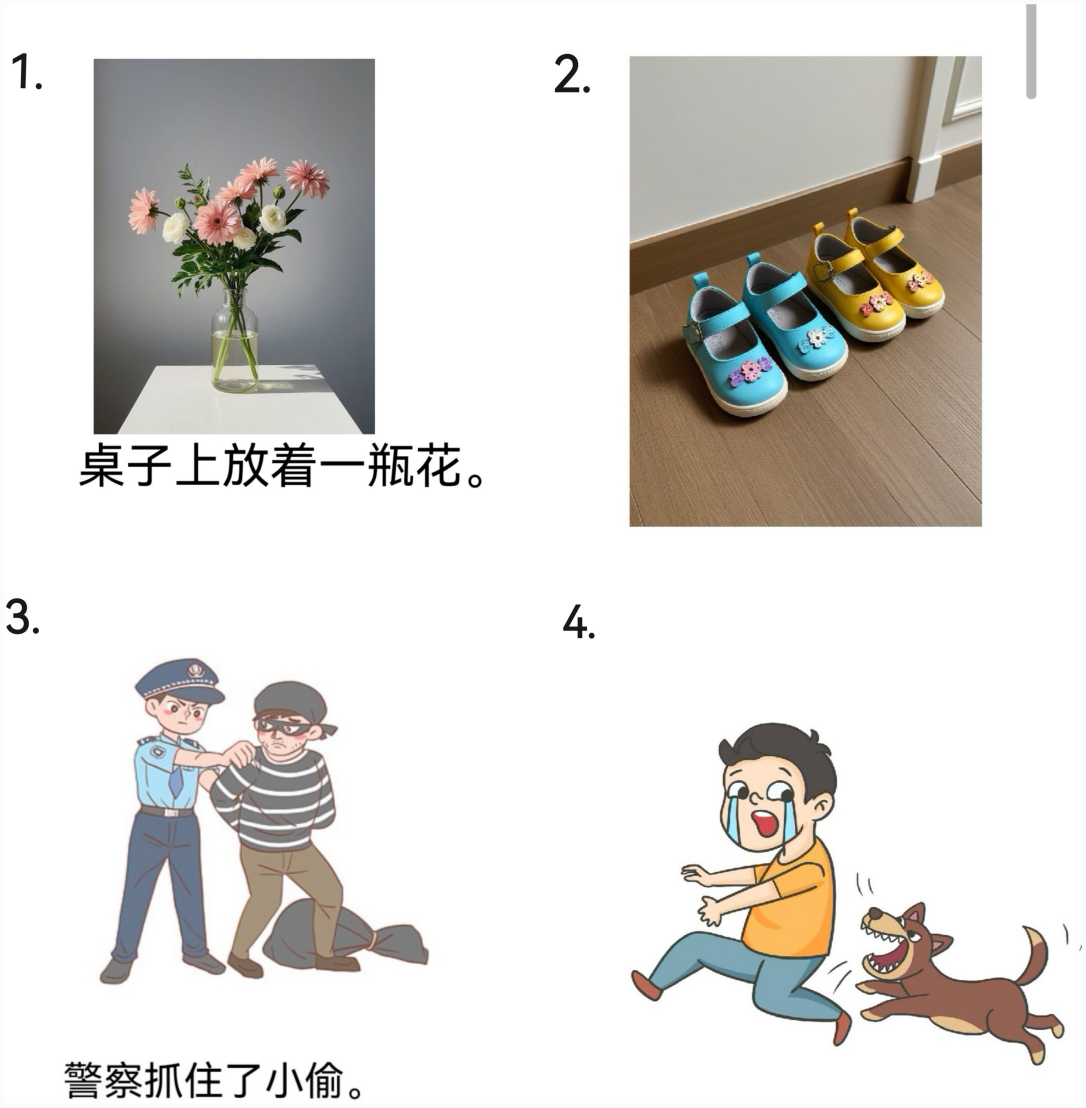
Example pictures from experiment 1. Picture 1 shows the prime pictures with sentences; picture 2 shows the target picture; picture 3 shows the filler prime pictures with sentence; picture 4 shows the filler target picture.

Fillers were designed to (i) reduce participants’ awareness of the prime–target manipulation and (ii) prevent habitual use of existential constructions. Among the 30 filler pictures, 12 were filler prime pictures with non-existential sentences to read aloud, and 18 were filler target pictures with no cue words to describe. Between any two experimental trials, 2–3 filler pictures were inserted at random. Figure 1 shows example stimuli.

#### 2.1.3 Procedure

Stimuli were presented using PsychoPy v2024.x on a Windows 10 PC with a 24″ LCD monitor (1920 × 1,080, 60 Hz; viewing distance ≈ 60 cm). Audio responses were recorded via a USB headset microphone (44.1 kHz, 16-bit), producing one .wav file per trial. After standardized instructions, participants completed a six-trial practice block (repeatable if needed). They were informed that the session would include three trial types: (a) prime pictures with a sentence to read aloud; (b) target pictures with no text to be described in one sentence mentioning all salient elements; and (c) filler pictures (some with sentences to read aloud; some to describe).

To ensure the procedure was transparent from the outset, participants completed a guided practice block and were required to correctly answer two comprehension questions about the trial sequence (prime reading vs. target description) before proceeding.

Trial order was randomized with the constraint that 2–3 fillers intervened between experimental trials. Prime types (EX-ZHE/EX-LE/EX-YOU) were counterbalanced across lists. Each prime trial began with a fixation cross (500 ms), followed by picture + sentence (3,500 ms) for aloud reading; an ISI of 500 ms preceded the next trial. Each target trial began with fixation (500 ms) followed by the target picture (no text), which remained on-screen until response or 8 s; a jittered ITI (800–1,200 ms) followed. Participants advanced trials with the spacebar. The experimenter did not interact with participants during the formal session. Total session duration was approximately 22 min.

PsychoPy logged trial-level information (participant ID, item ID, prime type, list, timestamps, response onset/duration, and audio filename) for subsequent coding and GLMM analyses.

### 2.2 Results

After collection, all recordings were transcribed verbatim and coded, yielding 1,260 target productions (105 participants × 12 targets). Responses were coded into four categories: 0 = non-existential or ill-formed output, 1 = EX-ZHE (Location + V + 着 + NP), 2 = EX-LE (Location + V + 了 + NP), and 3 = EX-YOU (Location +有 + NP). For descriptive purposes, existential responses (categories 1–3) occurred in 690/1260 trials (54.7%), whereas 570/1260 trials (45.3%) were coded as non-existential/other (category 0). Within the existential subset, EX-YOU responses were most frequent (405/1260 = 32.2% of all trials), followed by EX-ZHE (249/1260 = 19.8%) and EX-LE (36/1260 = 2.9%). This distribution indicates a strong baseline production bias toward EX-YOU in this population, consistent with the possibility that learners treat EX-YOU as a default existential template; critically, however, raw production frequency should not be conflated with priming magnitude and is therefore treated here as a baseline preference profile.

To quantify whether prime type and proficiency affected the likelihood of producing an existential sentence at all, we fitted a binomial generalized linear mixed-effects model (logit link) using glmer() in lme4. The dependent variable was Existential realization (1 = any existential response, i.e., EX-ZHE/EX-LE/EX-YOU; 0 = non-existential/other). Fixed effects included Prime Type (prime1 = EX-ZHE, prime2 = EX-LE, prime3 = EX-YOU), Proficiency (continuous), and their interaction; a random intercept for Participant was included.

As shown in Table 1, proficiency significantly increased the probability of producing an existential sentence, *β* = 0.242, SE = 0.089, z = 2.706, *p* = 0.007. Prime type also exerted a strong influence: relative to the model’s reference/contrast coding, prime1 reliably increased existential realization (*β* = 0.927, SE = 0.103, z = 8.958, *p* < 0.001), prime2 decreased it (*β* = −1.557, SE = 0.101, z = −15.396, *p* < 0.001), and prime3 increased it (*β* = 0.630, SE = 0.115, z = 5.478, *p* < 0.001). Importantly, Prime Type interacted with proficiency. Under prime1, the positive effect of proficiency was amplified (*β* = 0.445, SE = 0.099, z = 4.508, *p* < 0.001); under prime3, the proficiency slope was attenuated (*β* = −0.290, SE = 0.139, z = −2.100, *p* = 0.036); under prime2, the interaction was not reliable (β = −0.154, SE = 0.097, z = −1.590, *p* = 0.112). Together, these results indicate that (i) higher proficiency is associated with a greater likelihood of producing existential sentences overall, and (ii) primes modulate this likelihood in a construction-dependent manner, with proficiency acting as a selective moderator rather than a uniform amplifier.

**Table 1:** Summary of binomial GLMM results (DV: Existential realization).

Predictor	*β*	SE	*z*	*p*	OR	OR 95% CI (LL)	OR 95% CI (UL)
Intercept	0.475	0.091	5.245	<0.001	1.608	1.345	1.922
Proficiency	0.242	0.089	2.706	<0.05	1.274	1.070	1.517
Prime1	0.927	0.103	8.958	<0.001	2.527	2.065	3.092
Prime2	−1.557	0.101	−15.4	<0.001	0.211	0.173	0.257
Prime3	0.630	0.115	5.478	<0.001	1.878	1.499	2.352
Proficiency × prime1	0.445	0.099	4.508	<0.001	1.560	1.285	1.895
Proficiency × prime2	−0.154	0.097	−1.590	0.112	0.857	0.709	1.037
Proficiency × prime3	−0.290	0.139	−2.100	0.036	0.748	0.570	0.983

DV = Existential realization (1 = EX-ZHE/EX-LE/EX-YOU; 0 = non-existential/other). Coefficients are log-odds from a binomial GLMM (logit link) with random intercepts for Participant. Prime type was effect-coded (sum-to-zero contrasts): prime1 = EX-ZHE (“Location + V + 着 + NP”), prime2 = EX-LE (“Location + V + 了 + NP”), prime3 = EX-YOU (“Location + 有 + NP”). OR and 95% CIs were computed as exp(β) and exp(β ± 1.96 × SE).

For completeness, we also computed descriptive prime–target structural match rates within each prime condition. Because each prime type occurred in 420 target trials, the counts of structure-matching existential responses were 125/420 (29.8%) for EX-ZHE, 18/420 (4.3%) for EX-LE, and 251/420 (59.8%) for EX-YOU. These match rates provide a complementary view of construction-specific alignment tendencies, but they are conceptually distinct from the GLMM reported in Table 1, which models the binary outcome of existential realization (existential vs. non-existential).

### 2.3 Discussion

Experiment 1 investigated structural priming in L2 production of Mandarin existential constructions using a picture-description paradigm, targeting three static patterns: EX-ZHE (“Location + V + 着 + NP”), EX-LE (“Location + V + 了 + NP”), and EX-YOU (“Location + 有 + NP”). The GLMM results yield three key conclusions. First, structural priming was robust in the existential domain: prime type reliably shifted the likelihood of producing a prime-consistent target. Second, priming was strongly asymmetric across constructions: relative to the overall mean, EX-ZHE primes increased prime-consistent production (*β* = 0.927, *p* < 0.001), EX-YOU primes also increased it (*β* = 0.630, *p* < 0.001), whereas EX-LE primes produced a large suppression effect (*β* = −1.557, *p* < 0.001). Third, proficiency increased prime-consistent production overall (*β* = 0.242, *p* < 0.05), but this benefit depended on prime type: it was amplified following EX-ZHE primes (*β* = 0.445, *p* < 0.001), attenuated following EX-YOU primes (*β* = −0.290, *p* = 0.036), and not reliably modulated under EX-LE primes (*β* = −0.154, *p* = 0.112). Together, these findings establish existential priming in L2 Mandarin while also showing that priming is not a uniform “structural persistence” effect: its magnitude—and even direction—depends on construction-specific constraints and on learner proficiency, consistent with evidence that priming effects are heterogeneous across structures and contexts (Gries, 2005; Mahowald et al., 2016).

A central interpretive question is whether priming aligns with baseline preference or shows an inverse preference profile that has been linked to learning-based adaptation (Chang et al., 2006; Segaert et al., 2014). In the present study, preference was operationalized as baseline availability in targets (marginal construction rates across target responses). On this measure, EX-YOU was the most available construction, followed by EX-ZHE, whereas EX-LE was rare; prime-consistent production was also highest following EX-YOU primes and lowest following EX-LE primes. Thus, the overall pattern is best characterized as preference-consistent rather than inverse-preference. Crucially, however, EX-LE does not merely show “weak priming”; it shows active suppression (*β* = −1.557), indicating that construction-specific factors can override any tendency toward preference-based alignment. This framing also aligns with methodological cautions that priming-like repetition can be confounded with baseline distributions and verb–construction attraction, making it essential to treat preference and lexical bias as part of the explanatory landscape rather than as noise (Gries, 2005).

The asymmetric prime effects further suggest that availability and morphosyntactic commitment jointly constrain L2 priming in the existential domain. EX-YOU plausibly functions as a high-availability default existential schema for many learners; consequently, priming translates into overt alignment even at lower proficiency, and the proficiency slope is attenuated (β = −0.290). EX-ZHE, by contrast, requires integrating stative scene cues with the aspectual morphology 着, a mapping that plausibly strengthens with proficiency and yields the strongest moderation (*β* = 0.445). EX-LE imposes a commitment via 了 whose licensing conditions (telicity/scope/discourse integration) are often late-stabilizing in L2, making production more planning-intensive and uncertain; under such uncertainty, a prime may increase activation of an option without increasing selection of that option, leading to avoidance and the observed suppression effect (*β* = −1.557) rather than facilitation. A usage-based perspective provides a complementary explanation: if learners’ production is shaped by entrenched, experience-driven expectations linking lexical material and constructions, then high-availability templates (EX-YOU) will dominate competition, while lower-availability templates with narrower and more conditional distributions (especially involving aspectual marking) will be harder to retrieve under time pressure (Schmid, 2007).

Mechanism-level interpretation becomes clearer when residual activation and implicit learning are treated as complementary constraints rather than mutually exclusive explanations. On residual activation accounts, priming arises from temporary activation of recently used combinatorial representations, and persistence is most visible when the target option is accessible and low-cost to realize (Pickering and Branigan, 1998); this aligns with positive priming for EX-YOU and EX-ZHE and with the idea that EX-LE’s morphosyntactic commitment can block activation from converting into overt selection. On implicit learning accounts, priming reflects experience-driven updating of structural expectations (Chang et al., 2012; Reitter et al., 2011), often predicting stronger adaptation for less expected structures; the present data do not show a classic inverse preference pattern for EX-LE, but they suggest a boundary condition that is informative for learning-based views: when the mapping is highly uncertain, exposure may trigger conservative behavior (avoidance) rather than reinforcement. In contrast, EX-ZHE provides a more diagnostic case: the strong Prime × Proficiency interaction is consistent with the claim that updating effectiveness scales with representational stability, such that higher proficiency supports more reliable cue integration (stative construal + 着) and allows exposure to translate into measurable alignment.

The present findings also help situate existential priming within broader debates about what exactly is primed—surface order, hierarchical configuration, or form–meaning mapping. Cross-linguistic priming has been argued to transfer hierarchical configuration information, supporting abstract syntactic representations beyond discourse repetition (Desmet and Declercq, 2006). At the same time, evidence from lexical priming in parsing suggests that lexical representations encode detailed combinatory information that can guide syntactic processing, implying that “lexical” and “syntactic” influences are deeply intertwined (Novick et al., 2003). The existential domain likely sits at this interface: primes bias competition among constructional templates that differ in morphosyntax and mapping requirements, while proficiency changes the reliability with which learners can recruit these combinatory constraints.

Theoretical implications are threefold and speak directly to the reviewer’s concern about integrating results with existing theories and prior studies. First, L2 existential priming is reliable yet construction-sensitive: magnitude and direction depend on structural availability and morphosyntactic commitment, rather than being uniform across alternatives (Mahowald et al., 2016). Second, proficiency selectively strengthens alignment where form–meaning integration can stabilize (EX-ZHE), but does not globally amplify priming and does not eliminate the bottleneck associated with 了. Third, the co-occurrence of preference-consistent priming (EX-YOU) and construction-specific suppression (EX-LE) supports accounts that integrate online activation with experience-driven adaptation shaped by learners’ prior distributions (Chang et al., 2006; Gries, 2005; Pickering and Branigan, 1998; Schmid, 2007). Pedagogically, the results suggest decoupling “existence” from the EX-YOU default by strengthening the stative mapping and usage contexts that license EX-ZHE, and by introducing EX-LE in contexts with transparent telic endpoints and explicit discourse support to reduce uncertainty and increase availability.

Several limitations guide the next step and motivate Experiment 2. The present experiment targeted static scenes; dynamic/resultative contexts may alter licensing conditions, especially for EX-LE. Preference was operationalized from target distributions; future work can triangulate preference with corpus-based baselines and verb–construction attraction measures to address distributional confounds emphasized in corpus priming research (Gries, 2005). Finally, although lexical overlap was constrained, Experiment 1 does not localize lexical contributions to priming; manipulating overlap locus (e.g., head vs. non-head repetition) would better separate lexical facilitation from structural persistence and test whether overlap effects concentrate on structurally privileged elements, consistent with evidence that different constituents contribute asymmetrically to priming (Pickering and Branigan, 1998; Gagné, 2001). In sum, Experiment 1 demonstrates that structural priming in L2 Mandarin existential is robust but heterogeneous: learners readily align to EX-YOU, show proficiency-scaled alignment to EX-ZHE, and exhibit suppression for EX-LE, consistent with an account in which transient activation and adaptive updating operate under strong constraints from morphosyntactic commitment and representational stability.

## 3 Experiment 2

Experiment 1 demonstrated that structural priming in L2 Mandarin existential production is reliable but construction-sensitive: EX-ZHE showed clear priming that scaled with proficiency, EX-YOU behaved as a highly available default with attenuated proficiency modulation, and EX-LE showed suppression rather than facilitation. Building on these findings, Experiment 2 was designed to address a key mechanism-level issue left open by Experiment 1—namely, the extent to which L2 existential priming is strengthened by lexical repetition (the lexical boost) and whether any boost is concentrated on structurally privileged elements (e.g., the verb/head) rather than on other repeated constituents.

Specifically, by holding construction type constant (EX-ZHE) and varying which lexical element repeats, Experiment 2 provides a direct test of whether the facilitation observed in Experiment 1 reflects abstract structural persistence alone or is strengthened by lexically cued retrieval.

To isolate lexical overlap effects while holding the target construction constant, Experiment 2 focused on the existential pattern that showed both robust priming and clear proficiency scaling in Experiment 1, namely EX-ZHE (Location + V + 着 + NP). Using an online picture-description paradigm, we examined whether repeating different lexical elements between prime and target (verb vs. location noun vs. post-verbal NP) modulates priming magnitude, and whether such modulation differs by proficiency.

Accordingly, Experiment 2 addressed three questions:

(1) Does a lexical boost effect occur when different lexical elements are repeated between prime and target? (2) Is the lexical boost stronger for verb repetition than for repetition of other elements (location noun or post-verbal NP)?(3) Do priming and lexical boost effects differ as a function of proficiency (high vs. low proficiency)?

### 3.1 Method

#### 3.1.1 Participants

A total of 105 international students from the same population as in Experiment 1 participated in Experiment 2. (Participant characteristics, proficiency measurement, and compensation were identical to Experiment 1).

#### 3.1.2 Materials

Experiment 2 implemented four lexical-overlap conditions while keeping the prime construction constant (EX-ZHE). In each experimental trial, a prime picture was presented with an EX-ZHE sentence to be read aloud, followed by a target picture to be described using an existential sentence. Lexical overlap between prime and target was manipulated such that prime and target shared: (a) the verb, (b) the location noun, or (c) the post-verbal noun (NP). A non-repetition baseline condition (no lexical overlap) was included for comparison.

The verb set used in the existential sentences was identical to Experiment 1. A total of 72 picture sets were used, including 16 experimental prime pictures with sentences and 16 experimental target pictures. Target pictures displayed a cue word underneath that had to be incorporated into the spoken description. Specifically, there were four target sets per overlap condition: four sets with the same verb as the prime sentence, four sets with the same location noun as the prime sentence, four sets with the same post-verbal noun as the prime sentence, and four sets with no repeated word (baseline condition).

**Figure 2:**
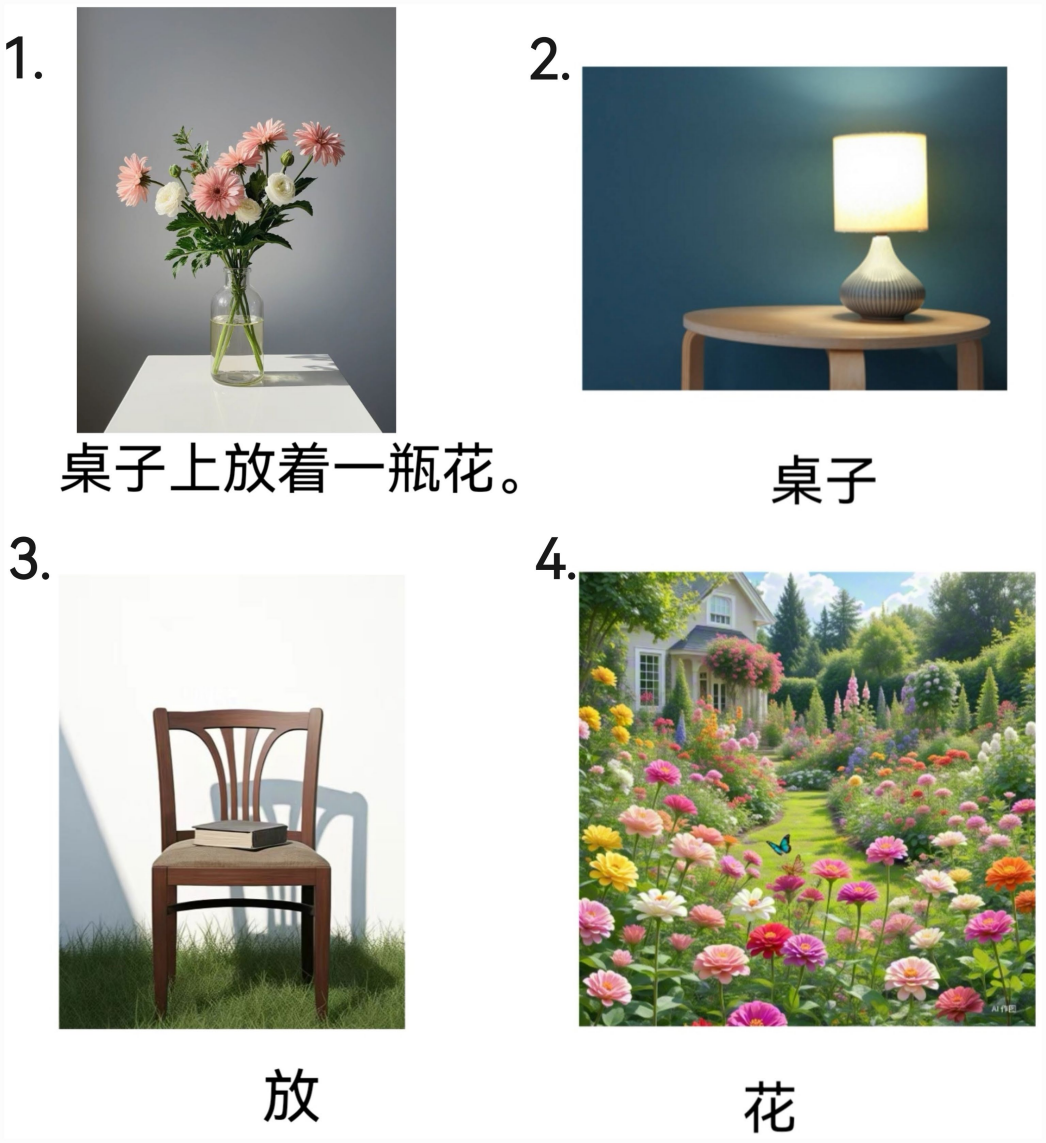
Example pictures from experiment 2. Picture 1 illustrates a prime trial with the sentence displayed below the picture. Pictures 2–4 illustrate target trials with a single-word cue. Picture 2 shows sentence-initial locative overlap (the target cue matches the prime’s locative word). Picture 3 shows verb overlap (the target cue matches the prime’s verb). Picture 4 shows post-verbal noun overlap (the target cue matches the prime’s post-verbal noun). The no-overlap (baseline) condition is not shown.

In addition, 40 filler pictures were included to mask the experimental manipulation and reduce strategic repetition. These fillers could only be described using non-existential sentences and comprised 20 filler prime pictures with sentences and 20 filler target pictures. Between each prime–target pair, 2–3 filler pictures were randomly inserted (Figure 2).

#### 3.1.3 Procedure

Stimuli were presented using PsychoPy v2024.x on a Windows 10 PC with a 24″ LCD monitor (1920 × 1,080, 60 Hz). Audio responses were recorded via a USB headset microphone at 44.1 kHz, with one .wav file saved per trial. After standardized instructions, participants completed three practice trials (repeatable as needed). They were informed that the session would include three trial types: (a) prime trials, in which they read aloud a sentence presented below a picture; (b) target trials, in which they described a picture using a single sentence that incorporated a cue word displayed above the picture (verb/location noun/post-verbal noun, or “—” in the no-overlap baseline condition) and mentioned all salient elements; and (c) filler trials, which were designed to be described using non-existential sentences.

Prime–target pairs were separated by 2–3 filler trials, and trial order was randomized. Each prime trial began with a fixation cross (500 ms), followed by the prime picture and sentence (3,500 ms). Each target trial began with fixation (500 ms), followed by the target picture with its cue; the display remained until a response was produced or 8 s elapsed, and was followed by a jittered inter-trial interval (ITI) of 800–1,200 ms. Participants advanced trials using the spacebar. No interaction occurred with the experimenter during the formal session. The session lasted approximately 30 min. PsychoPy logged trial-level information (participant ID, item ID, overlap condition, timestamps, and audio filename) for subsequent coding and GLMM analyses.

### 3.2 Results

All recordings were transcribed verbatim, yielding 1,680 target responses. To test lexical boost effects while holding the structural target constant, we focused on productions of the existential pattern EX-ZHE (“Location + V + 着 + NP”). Each trial was assigned to one of four prime–target lexical overlap conditions: Type 1 (verb overlap), Type 2 (post-verbal noun overlap), Type 3 (location-noun overlap), and Type 4 (no overlap; baseline). The dependent measure was a binary EX-ZHE realization indicator, coded 1 only if the target utterance realized EX-ZHE—i.e., a sentence-initial locative phrase followed by a stative verb marked with 着 and a post-verbal NP. All other outputs (including non-existential sentences, EX-LE/EX-YOU realizations, and ill-formed responses) were coded 0.

We fitted generalized linear mixed-effects models (logit link) in R. Fixed effects included overlap condition (Type 1–Type 4), proficiency (continuous), and their interaction. Random intercepts for participant and item were included. Model comparison was conducted to evaluate the contribution of fixed effects and the necessity of random slopes. Model 1 included only random intercepts. Model 2 added fixed effects of overlap condition, proficiency, and their interaction. Model 3 further added by-participant random slopes for overlap condition. Likelihood-ratio tests indicated that Model 2 provided a significantly better fit than Model 1 (χ^2^ = 63.183, df = 7, *p* < 0.001), whereas Model 3 did not improve fit relative to Model 2 (χ^2^ = 12.782, df = 9, *p* = 0.172). We therefore report estimates from Model 2.

**Figure 3:**
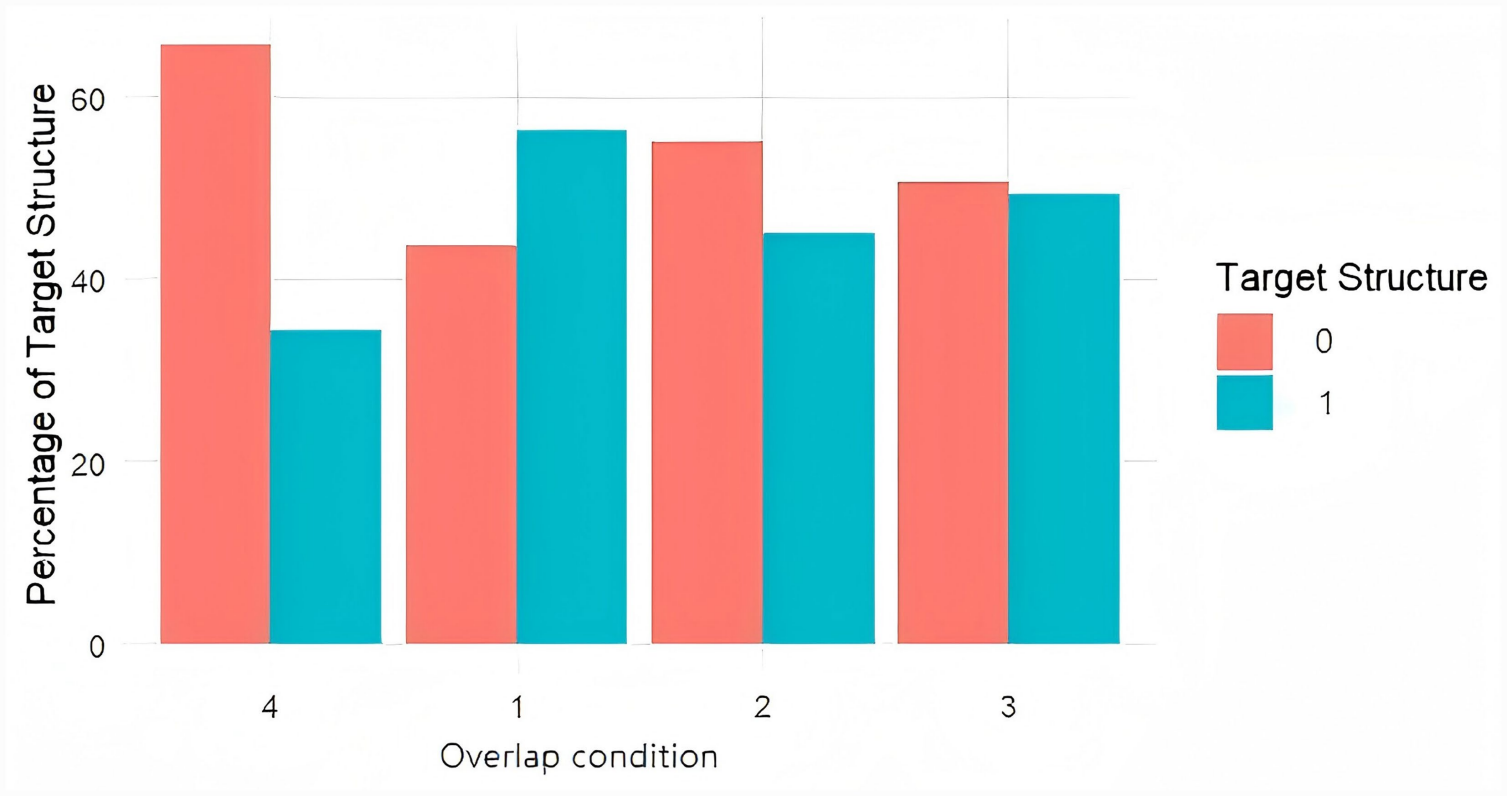
Effect of lexical overlap condition on EX-ZHE realization. The dependent variable was EX-ZHE realization (1 = the target utterance realized EX-ZHE: sentence-initial locative phrase + V + 着 + NP; 0 = any other output, including EX-YOU/EX-LE, non-existential descriptions, or ill-formed responses). Prime–target lexical overlap was coded as 1 = verb overlap, 2 = post-verbal noun overlap, 3 = location-noun overlap, and 4 = no overlap (baseline). Bars show the percentage of responses coded as EX-ZHE (1) versus other (0) within each overlap condition.

Overall, 45.6% of target responses were coded as EX-ZHE, indicating that nearly half of the targets matched the intended existential structure under the present constraints. Overlap condition significantly influenced the likelihood of producing EX-ZHE (Figure 3). Using the no-overlap baseline (Type 4) as the reference level, verb overlap (Type 1) produced a strong increase in EX-ZHE realizations (*β* = 1.044, SE = 0.152, *p* < 0.001), post-verbal noun overlap (Type 2) did not yield a reliable increase (*β* = 0.130, SE = 0.151, *p* = 0.391), and location-noun overlap (Type 3) significantly increased EX-ZHE realizations (*β* = 0.732, SE = 0.150, *p* < 0.001). Thus, lexical repetition enhanced structural alignment when overlap occurred at the verb (head) and, to a lesser extent, at the location noun, whereas repeating the post-verbal noun did not produce a measurable lexical boost.

Proficiency did not show a reliable main effect on EX-ZHE realization in Model 2 (*β* = 0.235, SE = 0.129, *p* = 0.069). In addition, the interaction between proficiency and overlap condition was not significant: proficiency × Type 1, *β* = 0.154, SE = 0.153, *p* = 0.316; proficiency × Type 2, *β* = −0.020, SE = 0.152, *p* = 0.895; proficiency × Type 3, *β* = −0.013, SE = 0.152, *p* = 0.930. These results suggest that, within the present design, lexical overlap effects were broadly similar across proficiency levels.

### 3.3 Discussion

Experiment 2 examined whether lexical repetition amplifies existential priming when the structural target is held constant, and whether any lexical boost depends on the repeated element and on proficiency. Building on Experiment 1, we selected EX-ZHE (“Location + V + 着 + NP”) as the target structure and manipulated prime–target lexical overlap at three loci—verb, location noun, and post-verbal noun—relative to a no-overlap baseline. The results provide clear evidence that priming in the existential domain is lexically sensitive, but that lexical boost is locus-dependent: relative to the baseline condition, verb overlap produced the largest increase in EX-ZHE realizations (*β* = 1.044, *p* < 0.001), location-noun overlap also produced a reliable increase (*β* = 0.732, *p* < 0.001), whereas post-verbal noun overlap did not yield a measurable boost (*β* = 0.130, *p* = 0.391). In addition, neither the main effect of proficiency (*p* = 0.069) nor its interactions with overlap condition were significant, indicating that the lexical boost pattern was broadly similar across proficiency levels in this design.

These findings refine the conclusions from Experiment 1 in three respects by clarifying where lexical support strengthens structural alignment. First, they show that the robust EX-ZHE priming observed in Experiment 1 can be further enhanced by repeating lexical material, consistent with the well-established lexical boost phenomenon in syntactic priming (Pickering and Branigan, 1998; Mahowald et al., 2016). Second, the strong effect of verb overlap aligns with accounts in which combinatorial frames are closely linked to verbal representations, making the verb a privileged retrieval cue for selecting the corresponding structural template (Pickering and Branigan, 1998). Third, the significant facilitation from location-noun overlap—in contrast to the null effect of post-verbal noun overlap—suggests that lexical boost in existential production is not determined simply by whether the repeated element is “head vs. non-head,” but by whether the repeated element occupies a cue-diagnostic planning position for committing to the EX-ZHE schema. In Mandarin existentials, the sentence-initial locative is part of the constructional “scaffold” that establishes the ground for existence predication; increasing its accessibility may therefore bias speakers toward initiating an existential frame, whereas repeating the post-verbal noun may contribute less to selecting the existential template because that noun can be accommodated by multiple competing descriptions (e.g., NP在PP, demonstratives, or other copular/locative statements). This interpretation is compatible with broader evidence that lexical and syntactic processes interact closely and that different constituents contribute asymmetrically depending on their role in guiding structural decisions (Gagné, 2001; Novick et al., 2003).

Mechanism-wise, the pattern supports a cue-sensitive view that is compatible with both residual activation and implicit learning, while also highlighting where each framework is most informative. Under residual activation accounts, lexical overlap boosts priming by increasing activation of the relevant combinatorial nodes; the stronger verb boost follows naturally because verbs are strongly tied to argument-structure and construction selection (Pickering and Branigan, 1998). The reliable location-noun boost can also be accommodated if the locative phrase serves as an early planning cue that pre-activates the existential scaffold, especially under time pressure in online production. Under implicit learning accounts, repeated mappings can strengthen expectations at the trial level, but the effectiveness of repetition should depend on whether the repeated element is a reliable cue for predicting the relevant structure; on this view, verb and locative overlap may provide more diagnostic evidence for selecting EX-ZHE than post-verbal noun overlap, which is less predictive because the same noun can appear in many alternative descriptions. In this sense, Experiment 2 complements Experiment 1 by showing that even when a construction is demonstrably proficiency-sensitive at the level of structural mapping (Experiment 1), lexical cueing can exert a relatively uniform, short-range influence on alignment once the target construction is within the learner’s productive repertoire.

The absence of proficiency moderation in Experiment 2 also helps integrate the two experiments into a coherent account. Experiment 1 showed that proficiency selectively moderated priming for EX-ZHE (i.e., proficiency mattered most where form–meaning integration and morphosyntactic commitment were non-trivial), whereas Experiment 2 indicates that lexical overlap effects on EX-ZHE realization are comparatively stable across proficiency levels. Taken together, these results suggest that proficiency is more strongly implicated in the structural/mapping component of existential production (stabilizing the EX-ZHE template and its licensing), while lexical overlap primarily provides transient retrieval support that can facilitate alignment even when proficiency differences remain. This interpretation is consistent with the idea that proficiency does not act as a global amplifier of priming, but instead modulates those components of production that depend on representational stability and cue integration.

Two design features qualify the interpretation and point to productive extensions. Because target trials included a cue word that participants were instructed to use, the observed lexical boost reflects the combined influence of (i) prime–target overlap and (ii) enforced retrieval of the cued element; future work can remove the cue or orthogonally manipulate cueing and overlap to isolate “pure” overlap effects. In addition, the dependent measure indexed EX-ZHE realization specifically; therefore, the null effect for post-verbal noun overlap should be interpreted narrowly as “no measurable increase in EX-ZHE commitment under noun repetition,” not as evidence that post-verbal nouns never facilitate production more generally. Finally, a fuller model of existential production would benefit from triangulation with distributional baselines (e.g., verb–construction attraction) to ensure that lexical overlap effects are not conflated with item-specific biases, a concern emphasized in corpus-based priming approaches.

In sum, Experiment 2 demonstrates a graded lexical boost pattern in existential priming when EX-ZHE is held constant: verb overlap yields the strongest facilitation, location-noun overlap yields a reliable but smaller facilitation, and post-verbal noun overlap does not differ from baseline, with no reliable modulation by proficiency. Combined with Experiment 1, the findings support a cost- and cue-sensitive view of L2 priming in which (i) construction-specific mapping stability determines where proficiency matters, and (ii) lexical overlap facilitates alignment primarily when it reinforces cue-diagnostic components of the existential template.

## 4 General discussion

This study investigated structural priming in L2 production of Mandarin existential sentences across two experiments and used the existential domain to probe how constructional availability, morphosyntactic commitment, lexical overlap, and proficiency jointly shape alignment. Experiment 1 established that priming is reliable in this domain but strongly construction-sensitive: EX-ZHE and EX-YOU primes increased the likelihood of producing matching targets, whereas EX-LE showed no reliable facilitation and, in our data, patterned as suppression rather than support. Proficiency increased target matching overall, yet its effect was selective rather than global—most evident for EX-ZHE, attenuated for EX-YOU, and absent for EX-LE—indicating that proficiency does not simply “increase priming,” but modulates priming where representational stability and form–meaning integration are most consequential.

Experiment 2 then isolated lexical overlap effects while holding the structural target constant by focusing on EX-ZHE (Location + V + 着 + NP) and manipulating prime–target overlap at three loci relative to a no-overlap baseline. The results revealed a graded lexical boost that was locus-dependent: verb overlap yielded the strongest facilitation, location-noun overlap also produced a reliable increase in EX-ZHE realization, and post-verbal noun overlap did not differ from baseline. Proficiency neither showed a robust main effect in this constrained design nor interacted reliably with overlap type. Taken together with Experiment 1, these findings suggest that once the EX-ZHE template is available for production, transient lexical support can promote alignment across proficiency levels, but the strength of that support depends on which lexical element is repeated and how diagnostic it is for initiating and maintaining the existential frame.

A coherent account emerges when residual activation and implicit learning are treated as complementary constraints operating under construction-specific costs. On a residual activation view, primes temporarily raise the availability of combinatorial frames, and the behavioral payoff of this activation depends on baseline accessibility and the morphosyntactic cost of committing to a form (Mahowald et al., 2016; Pickering and Branigan, 1998). This view naturally accommodates strong alignment for EX-YOU, plausibly a high-availability default for many learners, and proficiency-sensitive gains for EX-ZHE, whose production requires integrating stative scene cues with aspectual morphology 着. It also accommodates the weakness or suppression observed for EX-LE: if committing to 了 is planning-intensive or uncertain (e.g., due to telicity, scope, or discourse-licensing constraints), prime-induced activation may not translate into selection and can even increase avoidance. From an error-based learning perspective, trial-level evidence updates expectations over form–meaning mappings (Chang et al., 2012; Reitter et al., 2011). In this framework, evidence consistent with entrenched priors (EX-YOU) is integrated with minimal reweighting, whereas representationally unstable mappings (EX-LE) may fail to produce reinforcement and instead trigger conservative choice behavior; intermediate cases such as EX-ZHE can yield gains that scale with proficiency as cue integration stabilizes. The locus-dependent lexical boost in Experiment 2 further refines this mechanism story by showing that overlap facilitates alignment most effectively when it touches cue-diagnostic slots of the template: repeating the verb (a privileged retrieval cue for construction selection) provides strong support, whereas repeating the post-verbal noun—compatible with multiple alternative descriptions—provides little measurable advantage. The reliable benefit from location-noun repetition suggests that early-accessible “frame scaffold” material can also bias speakers toward initiating an existential template, even if it is not the syntactic head; this aligns with interface views in which lexical and syntactic influences interact and different constituents contribute asymmetrically depending on their role in guiding structural decisions (Gries, 2005; Novick et al., 2003).

These results also sharpen what the existential domain contributes to broader priming debates. First, priming effects are not uniformly distributed across constructions that are superficially similar; they are shaped by morphosyntactic commitment and the stability of form–meaning mappings in the learner’s grammar. Second, proficiency effects in priming are better treated as selective moderation—emerging most strongly where structural abstraction and cue integration are required—rather than as a general amplification factor. Third, lexical boost is not an undifferentiated “repetition helps” phenomenon: its impact depends on whether the repeated element serves as a reliable cue for selecting the relevant constructional template. The existential domain is therefore theoretically diagnostic precisely because it couples structural choice with aspectual marking and because its components (locative phrase, verb, post-verbal NP) differ in their capacity to cue template selection.

The findings also carry pedagogical implications for L2 Chinese instruction and production practice. These findings suggest that instruction may benefit from decoupling ‘existence’ from an EX-YOU default and from providing learners with structured opportunities to practice EX-ZHE and EX-LE under supportive semantic and discourse cues. We emphasize that these implications are hypotheses derived from controlled experiments and should be validated through classroom-based tasks and corpus-informed materials rather than taken as prescriptive replacements for naturalistic input. We do not argue that laboratory evidence should take precedence over usage-based resources; instead, controlled priming isolates mechanisms that can complement corpus distributions in guiding pedagogical task design. In practice, designing production tasks that emphasize verb-level planning—through carefully controlled verb sets and repeated exposure to verb–construction pairings—may yield stronger alignment than focusing on lexical availability of post-verbal nouns alone, consistent with the strong verb-overlap boost observed in Experiment 2.

Several limitations qualify the interpretation and point to future work. First, the materials targeted static existentials; dynamic or resultative contexts may impose different licensing requirements and could reshape priming magnitudes or directions, especially for EX-LE. Second, proficiency was grouped for presentation; future analyses that model proficiency continuously in hierarchical GLMMs can sharpen estimates of moderation and test non-linear relationships. Third, Experiment 2 required participants to use a cue word on target trials; accordingly, the observed boosts reflect the joint influence of overlap and enforced lexical retrieval. Future work should orthogonally manipulate cueing and overlap to isolate “pure” overlap effects and to determine whether locus-dependent boosts persist without cueing. A natural next step is triangulation: (i) estimate baseline distributions and verb–construction attraction for existential templates in learner corpora; (ii) test whether the same alignment asymmetries appear in semi-naturalistic dialogue tasks; and (iii) evaluate whether targeted classroom interventions (e.g., cue-rich EX-LE contexts) produce measurable gains in spontaneous production. Finally, item-level distributional factors (e.g., verb–construction attraction, frequency, and surprisal) can be incorporated to constrain alternative accounts and to address concerns raised by corpus-based priming approaches.

In sum, structural priming in L2 Mandarin existentials is reliable yet heterogeneous. Learners readily align to EX-YOU, show proficiency-dependent alignment to EX-ZHE, and show weak or suppressive alignment for EX-LE; lexical overlap further enhances alignment when it targets cue-diagnostic components of the template—most strongly the verb and, in this dataset, the location-noun scaffold—while post-verbal noun repetition provides little measurable benefit. This profile supports a cost- and cue-sensitive view of L2 production and clarifies where short-term alignment can be leveraged pedagogically and where representational instability limits its reach.

## Data Availability

The raw data supporting the conclusions of this article will be made available by the authors, without undue reservation.
